# Modified Supporting Materials to Fabricate Form Stable Phase Change Material with High Thermal Energy Storage

**DOI:** 10.3390/molecules28031309

**Published:** 2023-01-30

**Authors:** Chengbin Yu, Youngseok Song

**Affiliations:** 1 Research Institute of Advanced Materials (RIAM), Department of Materials Science and Engineering, Seoul National University, Seoul 08826, Republic of Korea; 2 Department of Fiber Convergence Materials Engineering, Dankook University, Yongin-si 16890, Republic of Korea

**Keywords:** thermal energy storage, phase change material, leakage, volume shrinkage

## Abstract

Thermal energy storage (TES) is vital to the absorption and release of plenty of external heat for various applications. For such storage, phase change material (PCM) has been considered as a sustainable energy material that can be integrated into a power generator. However, pure PCM has a leakage problem during the phase transition process, and we should fabricate a form stable PCM composite using some supporting materials. To prevent the leakage problem during the phase transition process, two different methods, microencapsulation and 3D porous infiltration, were used to fabricate PCM composites in this work. It was found that both microsphere and 3D porous aerogel supported PCM composites maintained their initial solid state without any leakage during the melting process. Compared with the microencapsulated PCM composite, the 3D porous aerogel supported PCM exhibited a relatively high weight fraction of working material due to its high porosity. In addition, the cross-linked graphene aerogel (GCA) could reduce volume shrinkage effectively during the infiltration process, and the GCA supported PCM composite kept a high latent heat (∆H) and form stability.

## 1. Introduction

Clean energy development is an essential line of research in both industry and academia due to the depletion of coal fuel and environmental pollution [1,2]. To replace traditional energy systems, water and solar energy harvesting is very attractive option [3,4]. The interminable sea water can generate hydrogen for energy fuel [5,6]. Hydrogen is regarded as one of the cleanest energies due to its lack of pollution, light density, and high heat of combustion [7,8]. Although the sea water technology for hydrogen generation is an unprecedented direction in the search to reduce environmental pollution and energy costs, air tightness restricts its further applications in the areas of aerospace technology and fuel cell development [9,10]. On the other hand, solar energy can be converted into various energy forms [11,12]. For instance, the solar-to-thermal energy conversion can be used for energy harvesting [13,14]. That is to say, heat energy, in some cases even waste heat, can generate electrical or mechanical energy [15,16]. To collect solar and heat energy effectively, substances such as thermal energy storage (TES) materials are generally employed. Various TES technologies have been utilized in industries and architecture. The collection of external thermal energy to maintain a relatively warm temperature can save a lot of energy and even reduce the environmental pollution [17,18]. That is to say, green energy is the true direction of energy technology, and we need a material with a high TES capacity and a low cost to use for green energy storage and harvesting. A thermal energy unit must maintain a large heat of fusion to absorb the external thermal energy effectively.

Phase change material (PCM) is being used in TES applications due to its high thermal density, capacity, and long-time durability [19,20]. The PCM can absorb and release a large amount of thermal energy during its phase transition process [21,22]. PCMs encompass various types of phase conversions, such as solid–liquid, liquid–gas, solid–gas, and solid–solid conversions. Based on the TES capacity during the phase transition process, the solid–liquid PCM shows a higher heat of fusion than any other type of PCM, and the solid–liquid PCM is most widely utilized in human life [23,24]). In addition, solid–liquid PCMs can be subdivided into organic, eutectic, and inorganic PCMs according to their crystal structures. Compared with the inorganic and eutectic PCMs, the organic PCM exhibits a suitable phase transition temperature and a high enough heat of fusion for storing a large amount of external solar energy [25,26]. The typical organic PCM contains polyethylene glycol (PEG), paraffin alcohol, and fatty acid with a high TES capacity [27,28]. However, leakage is a serious problem to solve for storing and releasing more thermal energy during the phase transition process [29,30]. To sustain the initial solid state of the PCM during the melting process, supporting materials need to be kept in the material system [31,32]. Thus, a supporting material entangled PCM composite has been introduced, and form stable PCMs have been fabricated [33,34]. Since the pure PCM can lose its initial solid shape during the melting process, it is necessary to wrap the surface of the working material to achieve form stability.

The microencapsulated PCM composite has a high form stability during the phase transition process [35,36]. PCM composites are composed of an internal core material and an external shell substance [37,38]. The pure PCM is encapsulated by the supporting material to construct a microsphere structure, thus resulting in a little thermal expansion during the melting process. Wang et al. has reported that polymethylmethacrylate (PMMA) could be used as a microencapsulated supporting material to fabricate a stearic acid/polymethylmethacrylate composite with form stability for high latent heat thermal energy storage (LHTES) [39]. Also, a leakage-proof PCM composite was prepared via the emulsification method using nano-cellulose as a supporting material [40]. The microsphere structures of PCM composites can prevent leakage during the phase transition process and sustain their initial state [41,42]. However, since the weight fraction of the pure PCM in the microencapsulated PCM composite decreases, the composite can store less external thermal energy than the pure PCM. To increase the total thermal energy absorption without leakage, a 3D porous aerogel has been utilized as an advanced supporting material [43,44]. The metal foam with a 3D porous structure can be applied to a cavity to enhance the high latent heat of the TES capacity for a phase change heat transfer as reported in Mehdi’s research [45,46]. Further research demonstrated that the metal foam can be utilized for the enhancement of high latent heat thermal energy storage (LHTES) units, which can be manufactured with a high thermal conductivity and permeability in a specified direction [47]. The high LHTES system with 3D porous foam was the best choice for infiltrating pure PCM into the internal structures and increasing the solidification time and the recovery rate [48]. That is to say, an aerogel structure with a high porosity can infiltrate a large amount of pure PCM, retaining a nearly 99 % weight fraction without any leakage.

In this study, we employed microencapsulation and porous aerogel infiltration methods to produce form stable PCM composites. The filler-surrounded composite was introduced to enhance the form stability of the composite. Since a 3D porous aerogel can hold pure PCM in the volume space, a graphene aerogel was integrated into the PCM composite. On the other hand, volume shrinkage is a problem in common graphene aerogels, which leads to a loss in the weight of pure PCM. The PCM composite TES capacity is proportional to the weight of pure PCM. The modified graphene aerogel with a high mechanical property and flexibility can reduce the volume shrinkage effectively to hold sufficient pure PCM in the infiltrating process.

## 2. Results and Discussion

### 2.1. Form Stable Microencapsulated PCM

**Figure 1:**
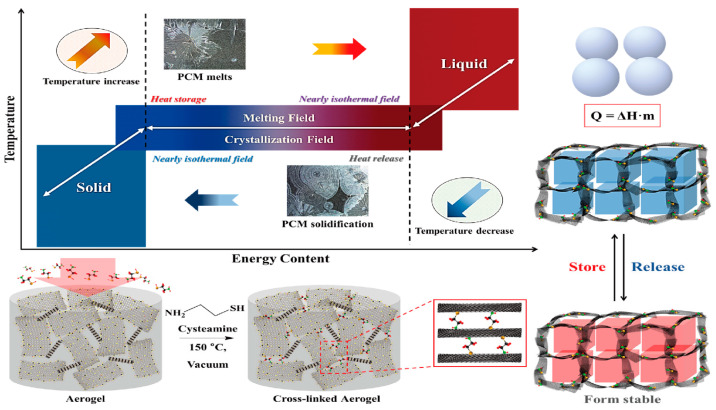
Schematic of PCM for energy storage and fabrication procedure for form stable PCM composite.

**Figure 2:**
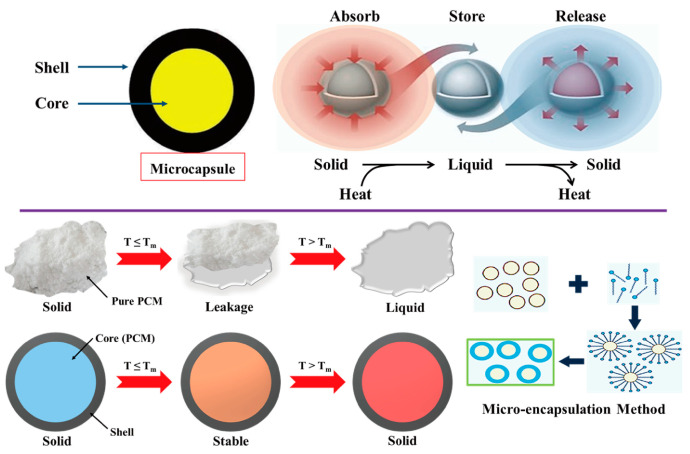
Illustration of working mechanism of microencapsulated form stable PCM composite.

**Figure 3:**
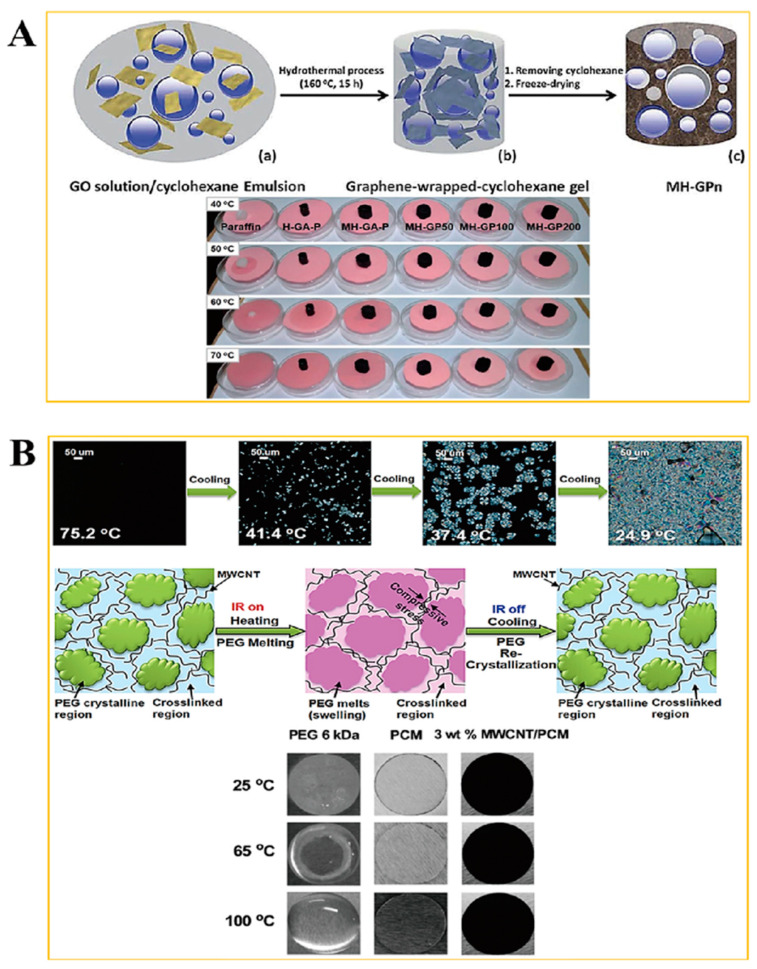
(**A**) paraffin microencapsulated PCM composite (**B**) cross-linked PEG form stable structure.

**Figure 4:**
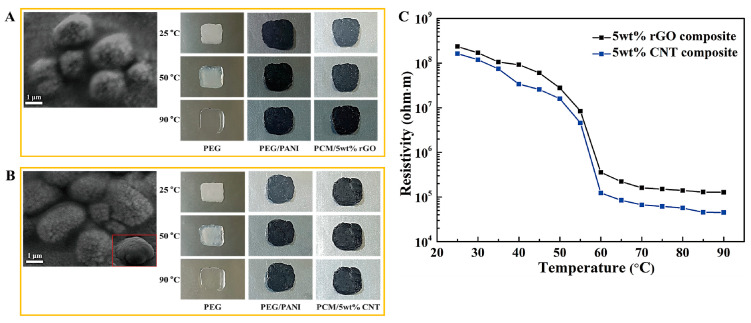
(**A**) PANI supported rGO/PEG composite, and (**B**) PANI supported CNT/PEG composite. (**C**) Variation of electrical resistivity under the change of temperature.

**Figure 5:**
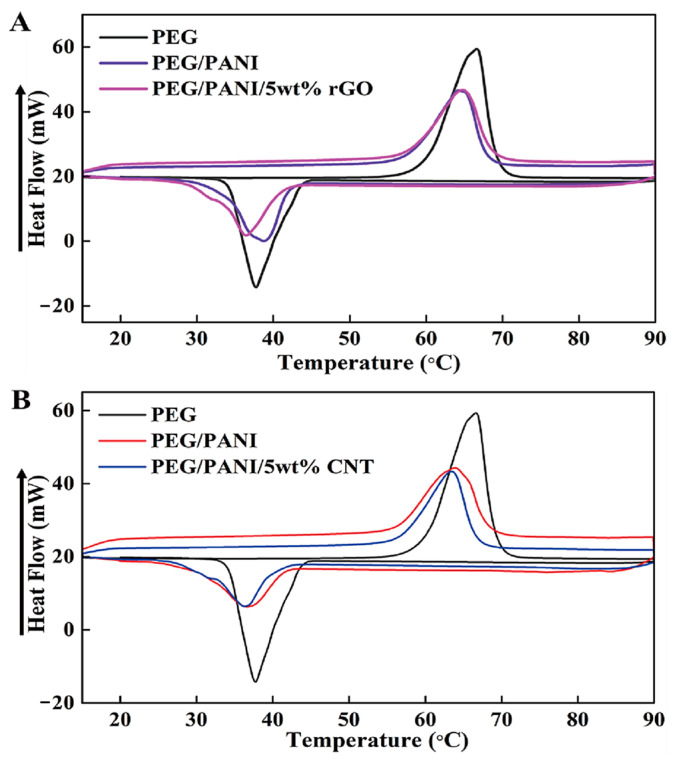
DSC results for the pure PEG and PEG/PANI composites filled with (**A**) rGO and (**B**) CNT.

Figure 1 shows the schematic of the PCM for energy storage and the fabrication procedure for a form stable PCM. The microencapsulated shell structure and 3D porous cross-linked graphene aerogel can hold plenty of pure PCM without leakage during the heating and cooling processes. The microcapsules in the PCM composites allow the melted PCM to be kept inside the microsphere due to the hydrophobicity. Because PCM is characterized by a high thermal energy storage (TES) capacity during the melting and cooling processes, we can utilize it as a nearly isothermal field for various TES applications. The thermal energy is related to the mass of pure PCM, and increasing the pure PCM weight fraction can promote the TES capacity significantly. Furthermore, the volume shrinkage of the 3D porous graphene aerogel during the pure PCM infiltration process decreases the amount of pure PCM, inhibiting the TES capacity. To reduce the volume shrinkage effectively, the cysteamine gas treatment method was employed in this fabrication process, and cysteamine was evaporated into the internal graphene aerogel structure so that the cysteamine would bond with the graphene oxide (GO) functional groups. Thus, a cross-linked network structure was generated that increased the mechanical property and flexibility of the aerogel to reduce the volume shrinkage. To compare the PCM composites made with two different kinds of supporting materials, we firstly will give a brief description of the microsphere PCM composite. The supporting shell materials encompass pure PCM to fabricate a form stable microencapsulated PCM composite, as shown in Figure 2. The shell material can surround the pure PCM to construct a core-shell structure which can keep the solid state without any leakage. Generally, the microencapsulated form stable PCM composite was fabricated using a solvent vapor method. The pure PCM exhibits a microsphere structure in the distilled water due to the hydrophobic property, while the shell material can be dissolved into the aqueous solution. The shell material can be absorbed into the surface of the microspheres of pure PCM gradually by evaporating the solvent. After completing the evaporation of distilled water, the microencapsulated form stable PCM composite was fabricated successfully. This demonstrated that the PCM composite can absorb or release a lot of thermal energy effectively during the phase transition process and sustain the initial solid state without any leakage. To improve the form stability after microencapsulation, graphene oxide (GO) was incorporated into paraffin wax in the cyclohexane solution [49]. The GO and paraffin wax embedded cyclohexane emulsion was vigorously stirred at 160 °C for 15 h (Figure 3A). The paraffin encapsulated PCM composite was obtained after removing the cyclohexane solvent and freeze-drying. The paraffin composites, especially MH-GA-P and MH-GP50, exhibited excellent form stabilities in the range of temperature. The modified PCM structure for maintaining the solid state was studied. The surface cross-linked PCM composite could prevent leakage even at 100 °C, as shown in Figure 3B [50]. This demonstrated that the shell material encapsulated PCM composites can sustain the initial solid state during the phase transition process. As in the previous work, the flexible polyaniline (PANI) was selected as a supporting material, and fabricated PEG/PANI composites were shown as Figure 4. The photographs of the PANI supported PEG composite are presented in Figure 4A,B [51,52]. The reduced graphene oxide (rGO) and carbon nanotube (CNT) were embedded as fillers to improve the electrical conductivity. The PANI entangled PEG composite was found to have a high form stability during the melting process. The microcapsule structure was analyzed by observing the SEM images and fully generated microsphere structures. The rGO and CNT fillers were dispersed at the surface of the microencapsulated PCM composites and can be easily connected to increase the electron movement under the temperature variations due to the volume expansion of the PCM composites. The results for the electrical resistivity are shown in Figure 4C, and the CNT embedded PCM composite had a higher electrical magnification than the PCM/rGO composite. The CNT with large aspect ratio exhibited an excellent electron carrier to promote the electrical conductivity during melting and cooling processes. A large portion of pure PCM was replaced with the supporting material, and thus the composite’s TES capacity was decreased significantly. Figure 5 shows the DSC results of the pure PEG and PEG composites prepared using the encapsulation process. The corresponding phase transition temperature and latent heat (∆H) are listed in Table 1. The melting point (T_mp_) of the pure PEG was 66.55 °C, and it absorbed plenty of external thermal energy, which can be confirmed by the results for latent heat (∆H). The melting latent heat (∆H_m_) was 179.44 J/g, while the cooling enthalpy (∆H_c_) showed 153.75 J/g. This indicated that a slight amount of stored energy was converted to kinetic energy due to the high viscosity. After the PEG was encapsulated with PANI, the melting and cooling points were expressed as 64.77 °C, and 38.78 °C, respectively. The PANI wrapped PEG composite still exhibited a similar phase transition temperature during the melting and cooling processes. However, the results for latent heat (∆H) were decreased significantly when compared with the pure PEG. The heat flow of the PEG composite was lower than that of the pure PEG due to the decrease in the weight fraction, and the latent heat (∆H) was not large enough to provide sufficient thermal energy. To increase the weight fraction of pure PCM, a supporting material with a 3D porous foam structure was introduced.

### 2.2. Aerogel Supported PCM Composite

**Figure 6:**
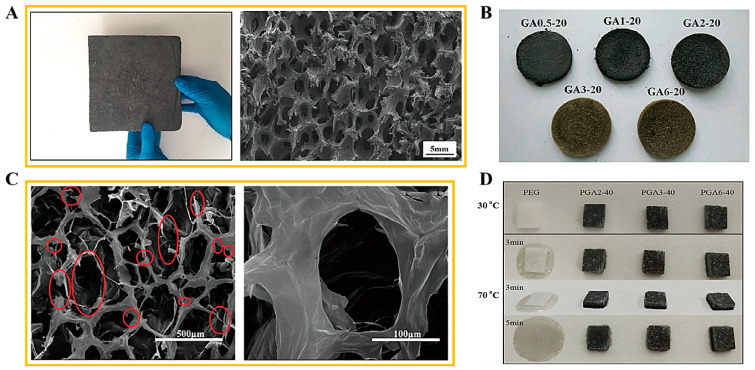
(**A**) graphene foam supported PCM composite, (**B**) GO/GNP aerogels with different concentration ratios, (**C**) SEM images of GO/GNP aerogel, and (**D**) photographs of pure PEG and PEG composite form.

**Figure 7:**
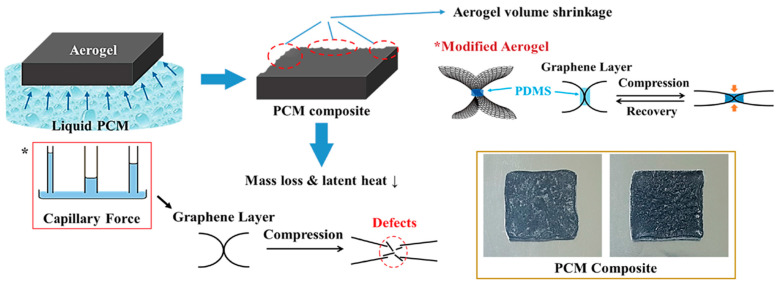
Schematic demonstration of the volume shrinkage of graphene aerogel.

A 3D porous foam structure was utilized for the PCM composite due to its high porous volume. Graphite foam was employed to fabricate a porous PCM sponge, as shown in Figure 6A [53]. The tremendous inter-porous structure was observed by the SEM imaging. The graphite foam supported PCM composites could retain their solid state during the phase transition process. The graphene aerogel was used as an appropriate porous supporting material due to its excellent chemical and thermal stabilities. The graphene nano-platelet (GNP) was embedded into the GO oxide to increase the mechanical properties. The GO aerogels with different concentration ratios are presented in Figure 6B. It was shown that the 3D porous structures were manufactured successfully [54]. Figure 6C shows the SEM images of the GO/GNP aerogel. It was found that the GNP embedded aerogel still maintained a high porous structure. This suggests that the porous GO/GNP aerogel can hold more PCM in the volume space than the microencapsulated structure. Thus, the GO aerogels supported PEG composites can maintain the solid state without leakage, as presented in Figure 6D. Although the GNP embedded GO aerogel was prepared successfully, the volume shrinkage of the 3D porous aerogel still its restricted further utilization. The capillary force that occurs during the infiltration process is demonstrated in Figure 7 [55]. The capillary force leads to a volume shrinkage of the graphene aerogel and defects in the internal skeleton. As the decrease in porosity causes a loss in weight in the final PCM composite, it can bring about a decrease in the latent heat (∆H). Thus, reducing the volume shrinkage is indispensable to fabricating a form stable PCM composite. One advanced method for reducing the volume shrinkage was fabricating a modified graphene aerogel with high flexibility infiltrating a PDMS solution into the internal graphene layers using the spray vapor treatment. The PDMS embedded graphene aerogel was obtained after evaporating the solvent at a high temperature. This modified graphene aerogel supported PCM composite has a high weight of pure PCM due to the reduction of the volume shrinkage. However, the mechanical properties were important for the form stability of a PCM composite which can sustain the solid state under external force at a high temperature. Therefore, a cross-linked GCA was introduced.

**Figure 8:**
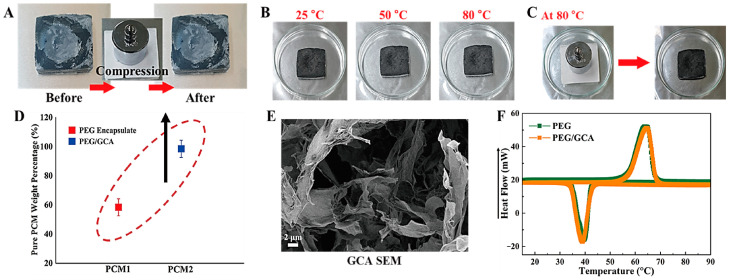
(**A**) Result of GCA flexibility test, (**B**) photographs of form stable test for PEG/GCA, (**C**) photographs of compression test for PEG/GCA, (**D**) PCM weight fraction of the microencapsulated PEG and PEG/GCA composites (**E**) SEM image of GCA, and (**F**) DSC results of the pure PEG and PEG/GCA composite.

A graphene/cysteamine cross-linked aerogel (GCA) was fabricated, and it provided a high flexibility under external compression, as shown in Figure 8A. Furthermore, the GCA supported PEG composite had an excellent form stability during the melting process and maintained the solid state under compression at 80 °C (Figure 8B,C) [56,57]. In addition, the GCA supported PEG composite can keep its initial shape without any leakage under the external force. This demonstrated that the GCA had the excellent mechanical properties and flexibility required to reduce the volume shrinkage and damage during the phase transition process. Based on the high porosity of the GCA, the GCA supported PEG composite exhibited a higher weight fraction of pure PEG than the PEG microcapsule, as shown in Figure 8D. The PEG/GCA had over 98% the weight fraction of pure PEG, while the microencapsulated PEG just showed 59%. From the results for the weight fraction, the 3D porous GCA can hold more working material than the microsphere PCM, and the GCA supported PEG composite can store or release a large amount of heat during the phase transition process. Figure 8E shows an SEM image of the GCA, and it confirms that the cross-linked GCA still kept a porous structure. The differences between the PEG/GCA and the pure PEG are shown in Figure 8F, and the results are listed in Table 2. The melting and cooling points (T_mp_, T_cp_) of the pure PEG were 65.72 °C, and 39.04 °C, respectively. The latent heat (∆H) of the pure PEG was increased up to 181.77 J/g, and the PEG/GCA showed a 178.90 J/g latent heat (∆H_m_) during the melting process. The PEG/GCA phase transition temperatures were close to those of the pure PEG due to the high weight fraction of the working material. As a result, the GCA supported form stable PEG composite can retain an amount of latent heat (∆H) that is large enough for TES applications.

## 3. Experimental Sections

### 3.1. Fabrication of Microencapsulated PCM Composite

The multi-walled carbon nanotubes (MWCNTs) embedded form stable PCM composite was fabricated from the synthesis of surface functionalized MWCNTs. The MWCNTs were functionalized using the 4-methoxyphenyl diazonium treatment. Polyethylene glycol (PEG) was utilized and 3.0 g of pure PEG, 122.5 mg of triphenylmethanetriisocyanate (TTI), and 1.5 mg of dibutyltin dilaurate (DBT) were mixed in distilled toluene and stirred for 6 h under a nitrogen atmosphere at around 85 °C to obtain a form stable PEG structure. After that, the functionalized MWCNTs were dispersed in *N,N*-dimethylformamide (DMF) via the ultrasonication method. The MWCNT solution and PEG toluene solution were mixed sufficiently using a Hielscher UP 400S probe ultrasonic sonicator. The mixture solution was ultrasonicated at 300 W for 30 min at 60 °C, and the modified MWCNT/PEG solution was heated to around 85 °C. The MWCNT/PEG was filtered by a grade 2 filter paper, and the resulting MWCNT/PEG nanocomposite film was finally obtained by evaporating the solvent under the vacuum state at 80 °C.

Referring to the previous research, the microencapsulated form stable PCM composite was demonstrated accessible and was further fabricated using a flexible supporting material. In this study, PEG was still selected as the pure PCM to be microencapsulated by polyaniline (PANI), which was synthesized by using 4 g of aniline monomer and 10 g of ammonium peroxydisulfate (APS). The mixture was poured into the dispersed water and stirred vigorously for 6 h at 80 °C. After evaporating the solvent, the melted PEG was wrapped into the PANI chains, and then a PEG/PANI composite was produced. Tests were conducted to observe the change in the electrical property during the phase transition process, which causes volume expansion due to the melting of the internal core material. The microsphere form stable PCM composite can maintain its initial solid state without leakage during the phase transition process. The graphene and CNT powders were used as fillers to be dispersed on the surface of the PEG/PANI composite. The filler weight fractions were controlled to 5 wt%, and we measured the change in electrical resistivity under different temperature fields.

### 3.2. Fabrication of PCM/Aerogel Composite

The paraffin/graphene form stable PCM composite was obtained using a homogeneous emulsion method. The graphene oxide (GO) was synthesized using the modified Hummers’ method and purified to make a GO powder [58,59]. The purified GO powder was then dispersed into distilled water to obtain a 20 mL GO aqueous solution, which was mixed with a cyclohexane solution including the paraffin max. The homogeneous GO/paraffin emulsion was acquired under a violent shake, and the emulsion was sealed in Teflon autoclaves to obtain a graphene/paraffin gel at a high temperature. After removing the heat source, the solidified graphene/paraffin gel was immersed into the distilled water at 90 °C for 1 h to evaporate the solvent, and the distilled water started to disperse the paraffin instead of cyclohexane. The graphene/paraffin hydrogel was fabricated and then subjected to the freeze-drying method to obtain a graphene/paraffin aerogel. The neat graphene aerogel was prepared using the hydrothermal method, and the PCM composite was fabricated by immersing the 3D porous graphene aerogel into the melted paraffin. It should be verified as a form stable PCM composite under the hydrothermal treatment. Based on a macro-porous pitch based graphite foam (PGF) with a high thermal conductivity, the synthesis of the PGF was started using a commercial sponge. The clean foam was utilized as a template was which immersed into a molten pitch, and sealed in an autoclave. The mold was filled with nitrogen gas and heated up to 500 °C for 5 h. The template foam was carbonized at 1000 °C in a furnace under nitrogen gas, and it was further graphitized at the same conditions, but with the temperature raised to 3000 °C. The PEG was melted at 100 °C under a vacuum state, and mixture of degassed hexamethylene diisocyanate (HDI) and PEG was stirred at 70 °C for 3 min and kept under vacuum at 80 °C for 6 h to obtain a form stable PEG/PGF composite.

However, the graphite foam required a complicated procedure, and we should modify the route of fabrication to a more convenient one. In the previous research, a GO aerogel was utilized as a supporting material, and this GO aerogel with different oxidation degrees was prepared from GO powder with an improved Hummers’ method [60]. After finishing the synthesis of the GO, the GO powder was dispersed into distilled water with different concentrations and these solutions were prepared under ultrasonication treatment. The dispersed GO solutions were poured into a cylindrical mold, and graphene aerogels were obtained using the freeze-drying method. The graphene aerogel was utilized as a supporting material to fabricate a form stable PCM composite, and PEG was melted into a liquid state at 90 °C. The form stable PEG composite was obtained using a vacuum impregnation method which immersed the graphene aerogel into the liquid PEG under the vacuum state for 3 h. The sample was fabricated after cooling at room temperature. Since the 3D porous graphene aerogel can hold a large amount of pure PCM in its internal volume space due to a high porosity, the graphene aerogel supported PCM composite exhibits a high weight fraction of working material with a high form stability. However, the volume shrinkage problem needs to be resolved to minimize the weight loss of working material during the infiltrating process. The capacity for thermal energy storage (TES) is related to the mass of pure PCM. It is necessary to prevent volume shrinkage in the fabrication process [61]. The oxidation of the graphene aerogel can generate additional functional groups for chemical reactions with cysteamine using the high temperature vapor method [62]. Thus, a cross-linked graphene aerogel (GCA) was utilized as an advanced supporting material and dipped into the melted PEG for 6 h with a vacuuming process. In this study, the modified graphene aerogel was fabricated via increasing the degree of oxidation of the GO. The GO powder was mixed with nitric acid (HNO_3_) and stirred at around 300 rpm for 6 h at 130 °C. After removing the excess nitric acid, a 160 mL potassium permanganate (KMnO_4_) solution was added to the mixture, and this suspension was kept for 2.5 h to further oxide the GO/HNO_3_ under the oil bath. To increase the degree of oxidation, 90 mL perchloric acid (HClO_4_) was poured into the mixture, and a citric acid solution was used to remove the excess KMnO_4_. The oxidized GO power was obtained using the freeze-drying method, and the modified Go aerogel was fabricated by adding the graphene nano-platelet (GNP). The oxidized GO/GNP aerogel was purified and the GCA was fabricated using the cysteamine vapor treatment. The oxidized GO/GNP was placed on the mini-chamber and put in a glass vial full of cysteamine solution at 150 °C for 72 h to produce a chemical reaction. The cysteamine gas can infiltrate into the internal GO skeletons and construct cross-linked chains sufficiently. The obtained GCA was placed on the liquid PEG at 80 °C for 6 h and, finally, the GCA supported PEG composite (PEG/GCA) was fabricated and solidified at room temperature.

### 3.3. Characterization

The surface morphologies of the PCM composite were observed by a field emission scanning electron microscope (FE-SEM, Merlin compact, ZEISS, Oberkochen, Germany) with an accelerating voltage of 5 kV. The phase transition temperature and latent heat (∆H) were obtained using a differential scanning calorimeter (DSC4000, PerkinElmer, Waltham, MA, USA) in a nitrogen atmosphere. The scanning temperature ranged from an initial 15 °C to a final 90 °C, with a rate of 10 °C/min. The electrical resistivity of the microencapsulated PCM composite was measured using a functional multi-meter (UT61, GuangZhou, China).

## 4. Conclusions

In this study, form stable PCM composites were prepared using the microencapsulation and 3D porous infiltration methods. The microsphere PEG composite was fabricated by wrapping with PANI. The PEG/PANI composite showed a high form stability during the melting process. The DSC result showed that a portion of the pure PEG was replaced with supporting material, and the weight loss caused a relatively low capacity for TES. Thus, the 3D porous foam structure was employed in the current study. The cross-linked GCA could reduce the volume shrinkage effectively during the infiltration process and sustain the solid state without any leakage under external force. Furthermore, the GCA supported PEG composite had a high PEG weight fraction and latent heat (∆H). The GCA can act as an advanced porous supporting material for thermal energy harvesting.

## Figures and Tables

**Table 1:** DSC characteristics of the pure PEG and PEG composites.

Samples	T_mp_ (°C)	T_cp_ (°C)	ΔH_m_ (J/g)	ΔH_c_ (J/g)
PEG	66.55	37.66	179.44	153.75
PEG/PANI	64.77	38.78	118.01	109.65
PEG/PANI/rGO	64.27	36.37	115.97	105.53
PEG/PANI/CNT	63.38	36.38	110.96	105.89

**Table 2:** DSC results of the GCA supported PCM composites.

Samples	T_mp_ (°C)	T_cp_ (°C)	ΔH_m_ (J/g)	ΔH_c_ (J/g)
PEG	65.72	39.04	181.77	160.02
PEG/GCA	64.84	39.01	178.90	159.22

## Data Availability

Not applicable.

## Sample Availability

Samples of the compounds are not available from authors.
